# Genomic analysis of an outbreak of Shiga toxin-producing Escherichia coli O183:H18 in the United Kingdom, 2023

**DOI:** 10.1099/mgen.0.001243

**Published:** 2024-05-21

**Authors:** David R. Greig, Orlagh I. Quinn, Ella V. Rodwell, Israel Olonade, Craig Swift, Amy Douglas, Sooria Balasegram, Claire Jenkins

**Affiliations:** 1 Gastrointestinal Bacteria Reference Unit, Public Health Microbiology, UK Health Security Agency, London, UK; 2 NIHR Health Protection Research Unit in Gastrointestinal Infections, University of Liverpool, Liverpool, UK; 3 Gastrointestinal Infections & Food Safety (One Health), Clinical & Public Health, UK Health Security Agency, London, UK

**Keywords:** genomic epidemiology, surveillance, outbreak, Shiga toxin-producing *Escherichia coli*, whole genome sequencing

## Abstract

In June 2023, UKHSA surveillance systems detected an outbreak of severe gastrointestinal symptoms caused by a rare serotype of Shiga toxin-producing *Escherichia coli*, STEC O183:H18. There were 26 cases aged 6 months to 74 years (42 % cases were aged 0–9 years), distributed across the UK with onset dates range between 22 May 2023 and 4 July 2023. The epidemiological and food chain investigations were inconclusive, although meat products made from beef mince were implicated as a potential vehicle. The outbreak strain belonged to sequence type (ST) 657 and harboured a Shiga toxin (*stx*) subtype *stx2a* located on a prophage that was unique in the UKHSA *stx*-encoding bacteriophage database. Plasmid encoded, putative virulence genes *subA*, *ehxA*, *saa, iha*, *lpfA* and *iss* were detected, however, the established STEC virulence genes involved in attachment to the gut mucosa (*eae* and *aggR*) were absent. The acquisition of *stx* across the global population structure of ST657 appeared to correspond with the presence of *subA*, *ehxA*, *saa, iha*, *lpfA* and *iss*. During the outbreak investigation, we used long read sequencing to characterise the plasmid and prophage content of this atypical STEC, to look for evidence to explain its recent emergence. Although we were unable to determine source and transmission route of the outbreak strain, the genomic analysis revealed potential clues as to how novel strains for STEC evolve. With the implementation of PCR capable of detecting all STEC, and genome sequencing for typing and virulence profiling, we have the tools to enable us to monitor the changing landscape of STEC. Improvements in the standardised collection of epidemiological data and trace-back strategies within the food industry, will ensure we have a surveillance system capable of alerting us to emerging threats to public health.

**Impact Statement**

Shiga toxin producing *Escherichia coli* (STEC) was identified as the cause of typical haemolytic uraemic syndrome (HUS) over forty years ago. The STEC pathotype is defined by the presence of a bacteriophage encoded Shiga toxin gene (*stx*) and comprises a diverse group of *E. coli*. The *stx*-encoding bacteriophage is a mobile genetic element, and we see a dynamic picture of acquisition, loss and re-acquisition constantly occurring across the population structure of *E. coli*. Acquisition of the *stx*-encoding bacteriophage in a compatible host, results in a stable STEC clone capable of proliferating and persisting in the animal reservoir with the potential to cause gastrointestinal disease and HUS in humans. In this study, we adopted a novel approach, using long read sequencing to characterise the plasmid and prophage content of an atypical STEC serotype O183:H18, that did not harbour the Locus of Enterocyte Effacment (LEE) pathogenicity island, to look for evidence to explain its recent emergence. In addition to the presence of *stx2a*, known to be associated with progression to HUS, we identified a plasmid carrying the putative pathogenicity factors, *espP*, *ehxA*, *saa*, *ihA* and *subA*. The *stx2a*-encoding prophage was unrelated to *stx*-encoding bacteriophage previously identified in the established UK STEC lineages and was located on a sparsely populated branch of the UKHSA bacteriophage phylogeny comprising a diverse set of phages encoding a variety of *stx* subtypes. STEC O183:H18 is a rare serotype in the UKHSA database, and together with our *stx*-encoding prophage analysis, we hypothesised that either this STEC serotype has been recently imported into the UK, or it is a domestic strain of *E. coli* that has recently acquired the *stx*-encoding phage from an external source.

## Data Summary

fastq files have been submitted to the National Centre for Biotechnology Information (NCBI). All data can be found under BioProject no. PRJNA315192 and strain-specific identifiers can be found in the Supplementary Table, available in the online version of this article.

## Introduction

*Escherichia coli* is a diverse species of bacteria found in humans, animals, food, and the environment. Acquisition of mobile genetic elements encoding virulence genes is a dynamic process constantly occurring across the *E. coli* population structure, resulting in the evolution of pathogenic lineages [1,2]. There are five well established pathotypes of *E. coli* that cause gastrointestinal infectious disease, including enteroinvasive *E. coli* (EIEC), enterotoxigenic *E. coli* (ETEC), enteropathogenic *E. coli* (EPEC), enteroaggregative *E. coli* (EAEC) and Shiga toxin-producing *E. coli* (STEC), each characterised by the presence of specific virulence gene markers [3]. The bacteriophage encoded Shiga toxin gene (*stx*) is the defining feature of STEC group. There are at least ten well established subtypes of Shiga toxin (stx1a, stx1c and stx1d, and stx2a-stx2g) [4]. A subset of STEC also have a pathogenicity island called the Locus of Enterocyte Effacement (LEE), the defining characteristic of the EPEC pathotype. The LEE facilitates attachment to the human gut mucosa and enhances pathogenic potential of *stx*-positive *E. coli*. The marker for the presence of the LEE is the *E. coli* attaching and effacing (*eae*) gene [5]. The defining characteristic of the EAEC pathotype is the presence of the aggregative adherence regulator gene (*aggR*). Although only a limited number of STEC serotypes also have been found to have *aggR*, these STEC/EAEC hybrid pathotypes have been associated with severe clinical outcomes [6,7].

Patients infected with STEC report a wide range of symptoms ranging from mild diarrhoea to severe, blood-stained diarrhoea, abdominal pain, fever and vomiting. Certain STEC types, most commonly those strains that harbour *stx2a* and/or *stx2d*, have the potential to cause haemolytic uraemic syndrome (HUS), a systemic condition characterised by thrombocytopenia, microangiopathic haemolytic anaemia, and acute kidney injury, that can be fatal [8,9]. Most STEC are zoonotic, and the animal reservoir is mainly ruminants, specifically cattle, sheep and goats but a wide range of wild and domestic animals and birds can be transiently colonised, and act as transmission vectors. Infection is transmitted to humans via direct contact with animals and/or their environment and by consumption of contaminated food and water. Common vehicles in foodborne outbreaks of STEC in the UK include undercooked beef and lamb meat [10,11], unpasteurised dairy products [12,13] and fresh produce contaminated by rainwater run-off or irrigation water containing animal excrement [14,15].

Historically, outbreaks of STEC in the United Kingdom (UK) and elsewhere around the world were most frequently attributed to STEC serotype O157:H7 [16,17]. Consequently, in some countries, including the UK, surveillance strategies focused on the development of a culture media, known as cefixime tellurite sorbitol MacConkey (CTSMAC) agar that was specifically selective for this serotype. Commercial gastrointestinal polymerase chain reaction (PCR) assays target the *stx* gene and therefore can detect all STEC serotypes, as the presence of *stx* defines the STEC pathotype. Over the last decade, the gradual implementation of GI PCR assays in local and regional diagnostic microbiology laboratories have provided evidence that a wide range of STEC serotypes are contributing to the clinical and public health burden of gastrointestinal infectious disease in England [9,18]. In recent years, outbreaks of non-O157 STEC have been linked to person-to-person transmission in nursery schools, contact with animals at petting farms, and foodborne outbreaks have been associated with unpasteurised dairy products and fresh produce (UKHSA in-house data, [19]).

In June 2023, UKHSA surveillance systems detected an outbreak of STEC O183:H18. This was a rare STEC serotype in the UK, and preliminary epidemiological analysis indicated patients were reporting severe clinical outcomes, and an incident management team (IMT) meeting was convened. Here, we describe the microbiological and epidemiological investigations and the application of sequencing data to determine the pathogenic potential of the outbreak strain and assess the risk to public health.

## Methods

### Microbiology

In England, all stool samples from hospitalised patients and community-acquired gastrointestinal (GI) infections are tested for STEC O157:H7 using cefixime-tellurite sorbitol MacConkey agar (CTSMAC) agar, and non-sorbitol fermenting colonies agglutinating with *E. coli* O157 antisera are referred to the Gastrointestinal Bacteria Reference Unit (GBRU), UK Health Security Agency (UKHSA) for confirmation and typing. Where local laboratories have implemented a commercial GI PCR that include primers targeting *stx*, all faecal specimens are tested for all STEC serotypes. *Stx*-positive samples are cultured on CTSMAC and/or Chromagar for STEC, and colonies exhibiting characteristics indicative of STEC may be referred to GBRU. Alternatively, the *stx*-positive faecal specimen may be referred to GBRU for PCR and culture [9].

### DNA extraction, Illumina sequencing and data processing

Genomic DNA was extracted via a Qiagen Qiasymphony and sequenced on Illumina HiSeq 2500 and NextSeq 1000 platforms. Post whole genome sequencing (WGS), genome data was processed through an in-house bioinformatics pipeline that determines serotype using GeneFinder (https://github.com/phe-bioinformatics/gene_finder). The *stx* subtype was determined by aligning putative reads to *stx* reference genes and detection of unique *stx*-subtype SNP positions [20]. Multi-locus sequence typing (MLST) was performed using a modified version of Metric Orientated Sequence Typer (MOST) (https://github.com/phe-bioinformatics/MOST) [36].

### Variant calling and cluster identification

Variant calling was performed by aligning sample reads (FASTQ) to an exemplar ST657 reference genome (NZ_CP027452.1) using BWA v0.7.3 [21], Samtools v0.7.17 [22], and GATK v2.6.5 UnifiedGenotyper [23] before import into SnapperDB v0.2.8 [24]. SNP (single nucleotide polymorphism) addresses are generated as part of SnapperDB v 0.2.8 [24] in for all isolates submitted to GBRU and are used to identify closely related strains and subsequently, clusters. As described previously, SNP address use a pairwise clustering approach to assign distance threshold levels that descend: Δ250, Δ100, Δ50, Δ25, Δ10, Δ5, Δ0. Each isolate within one level is no more than that many SNPs apart from the next isolate within the same level. For this study, clusters were defined as three or more isolates that differ in the zero SNP or five SNP level.

### Genomic and phylogenetic analysis of the Illumina sequencing data

A soft-core genome alignment of outbreak strains was generated from SnapperDB v0.2.8 of outbreak genomes. This alignment had recombination masked by Gubbins v2.00 [25] and a maximum-likelihood phylogeny was constructed using IQTree v2.0.4 [26].

Enterobase was interrogated to identify non-clinical isolates belonging to ST657 from around the world, using the Enterobase cgMLST algorithm to find unique strains that represented variable isolation sources, countries and HC-linkage clusters. Genomes were downloaded from the NCBI Sequence Read Archive (SRA) and genomes that were assigned serotype O183:H18 and passed internal UKHSA quality metrics were curated into a maximum likelihood phylogeny with UK strains using SnapperDB and a phylogenetic tree was constructed. The phylogeny (alignment length: 6949 bp) was viewed in ITOL and rooted on the most distant branch. Annotations are *stx* subtype and virulence gene presence, determined using Genefinder and in-house database supplemented with CGE virulence finder db. Cut off values for presence were >85% coverage and homology.

UKHSA and NCBI genomes were screened for the presence of putative virulence genes using the UKHSA in-house database and supplemented with variants from the Centre for Genomic Epidemiology *E. coli* virulence database (v2022-12-02 https://bitbucket.org/genomicepidemiology/virulencefinder_db/). Gene presence and *stx* profile were mapped onto the phylogenetic tree.

### Epidemiological investigations

In England and Wales, public health follow-up of cases of non-O157 STEC focuses on those that are infected with STEC that are positive by PCR for *stx2* and *eae*, because this pathogenic profile is significantly associated with the most severe clinical outcomes (Shiga toxin-producing *Escherichia coli*: public health management - GOV.UK [www.gov.uk]). Cases that are infected with STEC that are positive for *stx2* but negative for *eae* are not routinely administered an enhanced surveillance (ESQ) questionnaire (Shiga toxin-producing *Escherichia coli*: questionnaire - GOV.UK [www.gov.uk]). However, during this outbreak, Health Protection Teams were requested to administer ESQs to all patients microbiologically linked to the outbreak. In Scotland, ESQs are administered to all Scottish *E. coli* reference laboratory (SERL) confirmed cases of non-O157 STEC regardless of the *stx* profile. Following an initial review of the ESQ data, outbreak cases were re-interviewed using a trawling questionnaire to collect a more detailed food history 2 weeks prior to onset of symptoms.

The UK posted the outbreak on the European Communicable Disease Centre’s Epidemic Intelligence Information System sharing the WGS accession numbers to ascertain whether related cases had been seen elsewhere.

### Case-case analysis of exposures

Controls were selected from among sporadic STEC cases reported in the UKHSA national enhanced STEC surveillance system (NESSS) database. Controls were excluded if travel outside of the UK was reported in the 14 days before onset, if an enhanced surveillance questionnaire was not completed, or if they were younger or older than the age range of the cases associated with the outbreak (between 15 and 64 years). Food exposures were re-coded as binary responses. Binary variables were created for each food outlet, coded as one if premise was mentioned as the source of any food items. Odds ratios were calculated for each food outlet exposure comparing the odds of outbreak cases reporting the exposure with the odds of controls cases reporting the same exposure. *P*-values were adjusted for multiple testing. Multivariable logistic regressions were conducted for each exposure, adjusting for age and sex. This analysis was also repeated using all the STEC cases regardless of age and using logistic regression to adjust for age and sex.

### Nanopore sequencing and data processing

To investigate the *stx-*encoding prophages and genome structure, a subset of eight isolates were selected for Nanopore sequencing as described previously [27]. High-molecular weight genomic DNA was extracted using the Fire Monkey HMW DNA extraction kit (Revolugen), each DNA sample was barcoded using the rapid barcoding kit (SQK-RBK004). Sequencing was performed on a FLO-MIN106 flow cell and a MinION Mk1C (Oxford Nanopore Technologies) for 24 h.

Base calling of raw FAST5 data was performed using Guppy v6.5.7 FAST model. Read trimming, filtering and assembly was performed using Porechop v0.2.4 (https://github.com/rrwick/Porechop), Filtlong v0.2.0 (https://github.com/rrwick/Filtlong) and Flye v2.9 [28] respectively. Assemblies were corrected using a four-step process, firstly using Racon v1.4.20 [29] for four rounds followed by Medaka v1.0.3 (Oxford Nanopore Technologies) using a STEC specific trained model (https://github.com/gingerdave269/stec_medaka_model) with self-alignments generated from minimap2 v2.24 [30] and Samtools v1.10.0. Thirdly, the Illumina reads of each sample were processed using Pilon v1.23 [31] with BWA MEM v0.7.17 [21] and Samtools v1.10.0 [22] and finally a round of Racon v1.4.20 [29]. As the chromosome from each assembly was circularised and closed, they were re-orientated to start at the *dnaA* gene (GenBank accession no. NC_000913) from *E. coli* K-12, using the --fixstart parameter in Circlator v1.5.5 [32].

### Prophage detection, plasmid typing, chromosome and Stx-encoding prophage comparisons

*Stx-*encoding prophages were detected and extracted using PhageBoost and Propi v0.0.1 [33] re-annotated using PGAP (build6771) [34], aligned and compared using Clinker v0.0.27 [35]. The *stx*-encoding prophages were also compared to a UKHSA in-house database of *stx-*encoding prophages (totalling 404) in a pairwise fashion using Mash V2.2 [36]. Mash was used to sketch (sketch length=1000, kmer length=21) all extracted prophages and the resulting pairwise Jaccard distances between the prophages was used to construct a neighbour-joining tree and visualised using FigTree v1.4.4.

### Plasmid detection and characterisation

Plasmids were identified in Nanopore-based assemblies as closed circular contigs with a single plasmid replicon. Plasmid replicon detection was performed using PlasmidFinder v.2.135 with the Enterobacteriaceae, minimum identity=90 % and minimum coverage=90 % parameters set. Annotations from PGAP (build6771) [34], were used in conjunction with BRIG v0.95 [37] to visualise the IncFIB plasmid in the dataset.

### Data deposition

All FASTQ and assemblies were submitted to the NCBI. Illumina FASTQ accessions, Nanopore FASTQ accessions and assembly accessions (chromosome and plasmid) can be found under BioProject: PRJNA315192. For full details please see Table S1.

## Results

### Geography, age, sex and clinical outcome

**Fig. 1.**
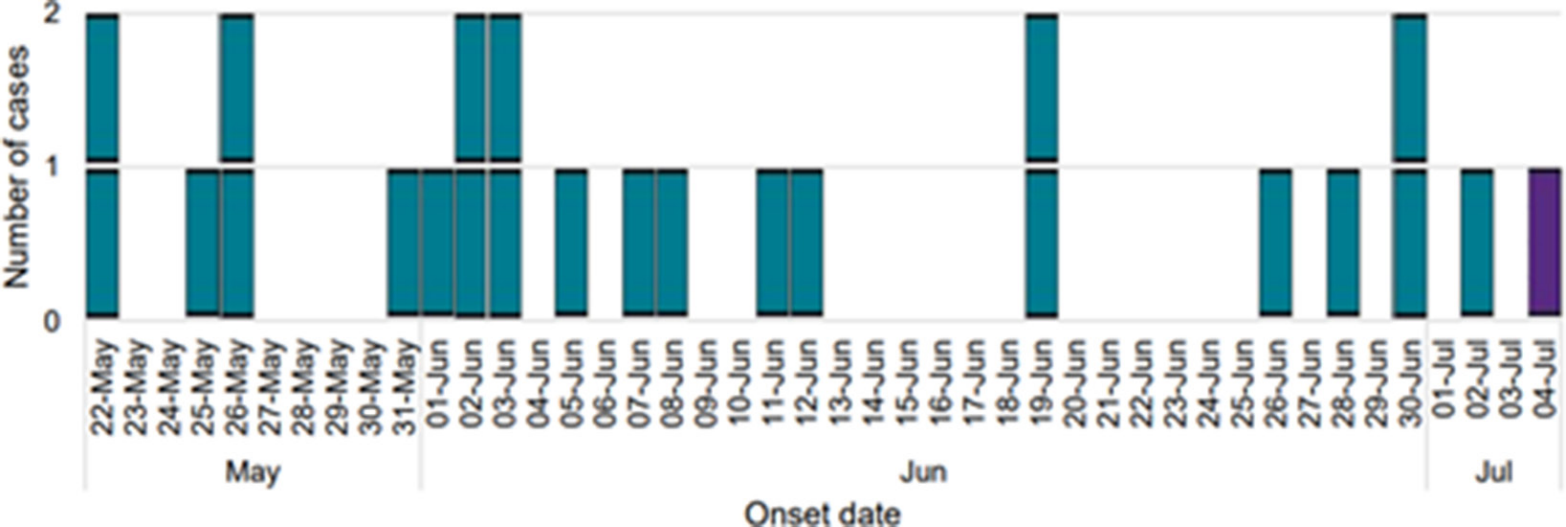
Temporal distribution of cases based on onset date (*n*=24). Onset data was not available for 2 cases.

**Fig. 2.**
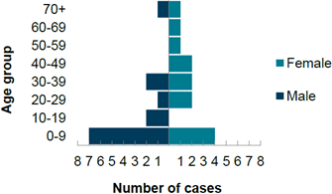
Age and sex distribution of cases by 10 year age group (*n*=26).

There were 27 confirmed cases and one probable case linked to the outbreak. These 28 cases were distributed across all nine UKHSA regions in England (*n*=21), as well as Northern Ireland (*n*=3), Scotland (*n*=2) and Wales (*n*=2) (Table 1). The UKHSA region with the most reported cases was the South East of England (Table 1). Three cases were unavailable for interview and another case was an asymptomatic secondary contact of a primary case. For the remaining cases (*n*=24) dates of onset of symptoms ranged between 22 May 2023 and 4 July 2023 (Fig. 1). The age range was from 6 months to 74 years with a median of 17 years, with the highest number of cases (*n*=11, 42 %) being seen in the 0–9 years old age group. There was an equal number of female (*n*=13) and male cases (*n*=13) (Fig. 2).

**Table 1.** Distribution of cases by region and country

Region/Country	No. of cases (%)
England	21 (81)
East Midlands	1 (4)
East of England	3 (12)
London	2 (8)
North East	1 (4)
South East	7 (27)
South West	2 (8)
West Midlands	2 (8)
Yorkshire and Humber	1 (4)
North West	2 (8)
Northern Ireland	2 (8)
Scotland	2 (8)
Wales	1 (4)
Total	26

For those cases where information was available (*n*=24), the majority reported bloody stools (*n*=17, 71 %), and eight (33 %) cases sought hospital care (Table 2). To date there has been one case of HUS, and one reported death in a case associated with this outbreak, however STEC was not listed as causal or contributory on the death certificate and the potential contribution of their STEC infection to this death is uncertain.

**Table 2.** Clinical information including reported symptoms

Clinical information	No. of cases (%)
Diarrhoea	23 (96 %)
Blood in stool	17 (71 %)
Nausea	12 (50 %)
Vomiting	11 (46 %)
Abdominal pain	21 (88 %)
Fever	11 (46 %)
Admitted to hospital	6 (25 %)

### Travel and food histories

Exposure information was collated from the ESQs available for 24 of the cases, 19 resident in England, two resident in Scotland, one resident in Wales and one resident in Northern Ireland. Two cases (11 %) reported travelling outside the UK, one case reported travel to mainland Europe (3 days prior to onset) and the other reported travel to Ireland (resident in Northern Ireland) (7 days prior to onset).

Food exposure data was collated from the ESQs, and food items reported by more than 50 % of cases reported cooked chicken (68 %), cooked beef (55 %), pasteurised milk (68 %) and hard cheeses (77 %).

Ten of the confirmed cases were available for follow-up and completed an additional trawling questionnaire with an interviewer. These confirmed cases range from 2 to 60 years, with a median of 20 years old, and are resident in England (*n*=7), Scotland (*n*=2) and Northern Ireland (*n*=1). Efforts to complete a trawling questionnaire with the remaining cases were confounded by the cases either not being available or willing to engage further. From the trawling questionnaires, the commonly reported food items were strawberries (70 %), pasteurised milk (80 %), hard cheeses (100 %), chicken (100 %), and beef (100 %). All commonly reported food items were investigated, although chicken and pasteurised dairy products were deemed to be unlikely to be vehicles for transmitting STEC infection, as no previous STEC outbreaks linked to these food items had been previously recorded in the UK (UKHSA in-house data). In contrast, cattle are well established as an animal reservoir for STEC, and undercooked beef meat products, particularly beef burgers and beef mince, are often implicated in foodborne outbreaks of STEC [10,11, 38]. All ten cases that completed a trawling questionnaire reported consumption of either beef burgers, beef mince cooked at home or other beef mince products.

The colleagues from the Food Standards Agency (FSA) initiated a food chain investigation to ascertain common beef suppliers and processers in order to identify the origin of the beef. However, the complexity of the beef industry supply lines hampered progress. Food chain investigations were de-escalated once the outbreak was over, and the source of the contaminated food was never confirmed.

### Virulence genes

The outbreak strain belonged to serotype O183:H18, ST657, and had the Shiga toxin subtype, *stx2a*, but did not have either of the two established STEC adherence factors, *eae* or *aggR*. In additional the Shiga toxin, genome interrogation of short read sequencing data identified the presence of additional toxin associated genes, specifically the subtilase cytoxoin gene, *subA* and the enterohaemolysin gene, *ehxA*. Although *eae* and *aggR* were both absent, other attachment/adhesin-associated genes were detected, specifically STEC autoagglutinating adhesin gene, *saa*, the *IrgA* homologue adhesin, *iha* and the long polar fimbriae gene *lpfA*. Furthermore, *iss* associated with serum resistance and *espP*, an extracellular serine protease, were also identified.

### Analysis of prophage

**Fig. 3.**
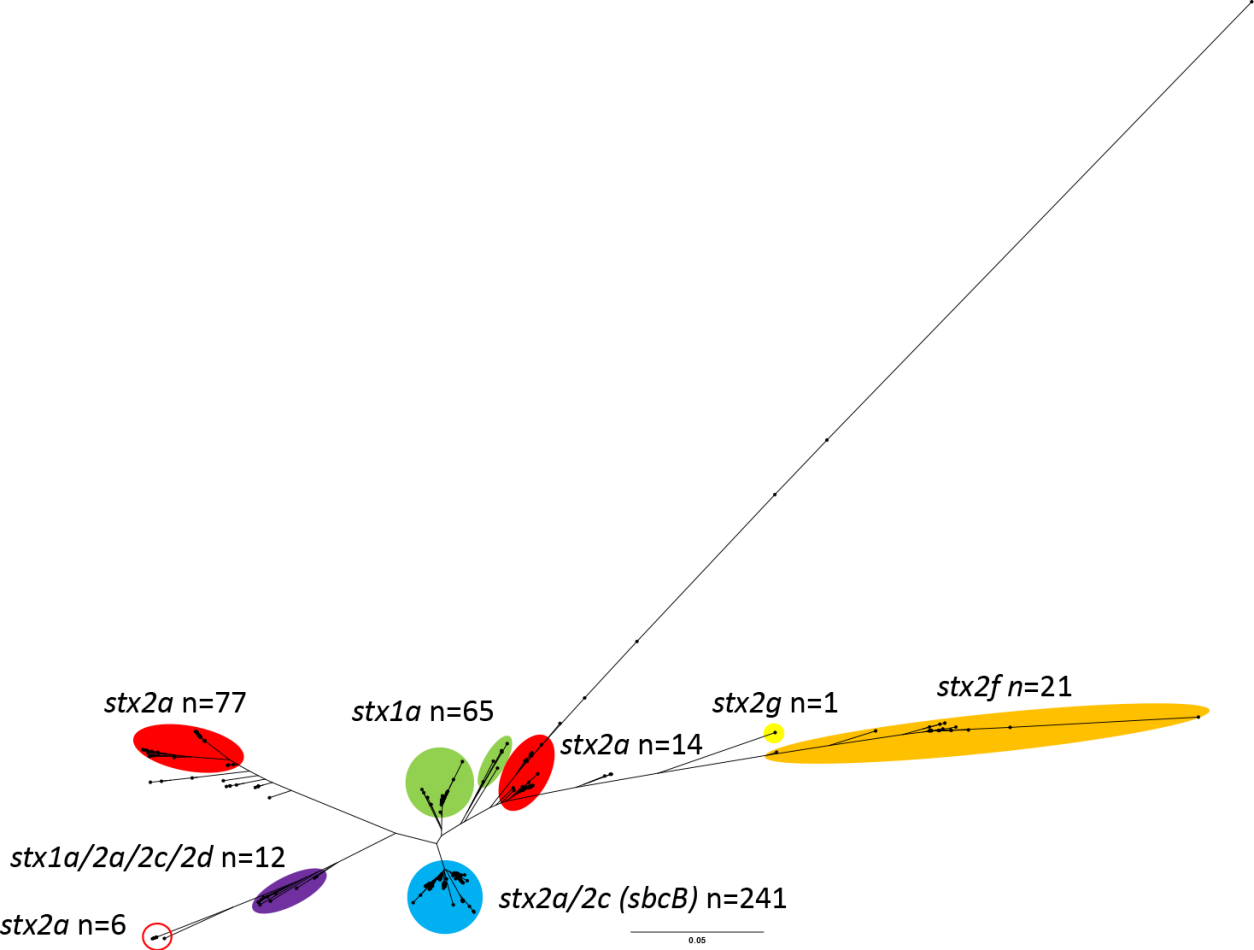
Comparison of *stx-*encoding prophages (*n*=404) via Jacard distances from UKHSA archives. The *stx2a-*encoding prophages found in the STEC O183 outbreak strain are circled in red.

Processing and analysis of the long read sequencing data enabled us to generate complete assemblies of the outbreak strain, and then excise, characterise and compare the *stx*-encoding prophages with STEC prophage sequences in the UKHSA archive. The sequence of the *stx2a*-encoding prophage in the STEC O183:H18 outbreak strain was uniquely different to other *stx2a*-encoding prophages previously identified in the common UK domestic STEC lineages and was located on discrete clade comprising 18 diverse prophages encoding *stx1a*, *stx2a, stx2d* or *stx2d* (Fig. 3 and Table 3). Although phylogenetically distinct, the most closely related prophage encoded *stx1a* and was excised from an isolate also belonging to the same ST as the outbreak strain, ST657, from a case reporting recent travel to Ethiopia (Fig. S2 and Table 3). The remaining highly diverse *stx*-encoding prophages in this clade were excised from strains of STEC belonging to four different clonal complexes (CCs), including CC165, CC11, CC29 and CC17. These strains were isolates from 11 individuals, six (55 %) reported travelling outside the UK prior or onset of symptoms, and five (45 %) were diagnosed with STEC-HUS.

**Table 3.** Epidemiological data linked to the isolates exhibiting *stx*-encoding phage located within the same clade as the *stx*-encoding phage harboured by the outbreak strain; ST Sequence type; CC clonal complex

SRA accession	Year	Sex	Age	HUS	Travel	STEC serotype	ST	CC	EAE	*Stx* subtype
SRR14347917	2021	M	1	Y	No travel history available	O26:H11	29	CC21	+	*stx2a*
SRR12635475	2020	M	11	Y	France	O80:H2	301	CC165	+	*stx2d*
SRR12362236	2020	M	1	Y	No travel reported	O45:H2	301	CC165	+	*stx2a*
SRR11215181	2020	M	15	N	No travel reported	O103:H2	17	CC17	+	*stx1a,stx2a*
SRR10386573	2019	M	2	N	No travel reported	O80:H2	301	CC165	+	*stx2a*
SRR11361822	2019	M	82	N	France	O26:H11	16	CC21	+	*stx1a,stx2a*
SRR8368697	2018	F	85	Y	No travel reported	O80:H2	301	CC165	+	*stx2d*
SRR7850110	2018	M	16	N	Japan	O157:H7	11	CC11	+	*stx2a,stx1a*
SRR4787009	2016	M	34	N	Italy	O157:H7	11	CC11	+	*stx2a,stx2c*
SRR3574263	2016	F	1	Y	Portugal	O55:H9	301	CC165	+	*stx2d*
SRR7191554	2015	F	29	N	Ethiopia	O61:H34	657	CC657	−	*stx1a*

### Phylogenetic analyses of ST657

**Fig. 4.**
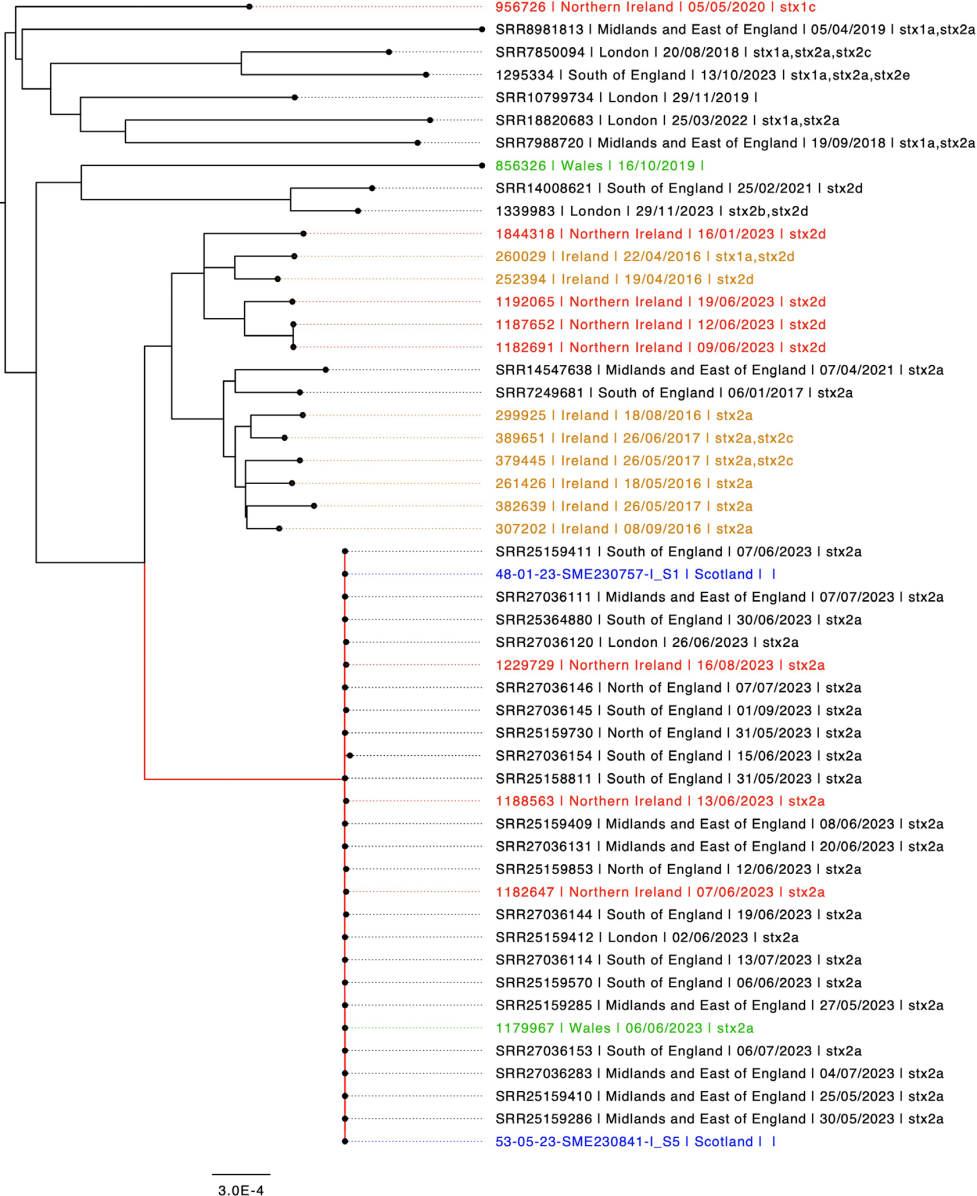
Maximum-likelihood phylogeny of a soft-core alignment of ST657 genomes coloured by country showing Country/Region, Sample date and *stx* subtype. Outbreak clade highlighted in red.

Interrogation of the UKHSA WGS data archives identified a total of 51 isolates belonging to ST657, of which 27 were linked to the outbreak cluster (highlighted in red in Fig. 4) and 24 were from sporadic cases (Fig. 4). Of these, 24 were from Ireland or Scotland or Northern Ireland, or Wales (Fig. 4). The outbreak isolates belonged to a distinct, closely related cluster (highlighted in red in Fig. 4) comprising of 27 isolates with an average SNP distance of fewer than one SNP and greater than 258 SNPs from the other isolates belonging to ST657. With the exception of the outbreak cluster, the ST657 phylogeny was sparsely populated with a diverse group of strains. Most of the sporadic isolates had either *stx2a* (*n*=13/24, 54%) or *stx2d* (*n*=8/24, 33%), and none of the isolates had *eae* or *aggR*.

**Fig. 5.**
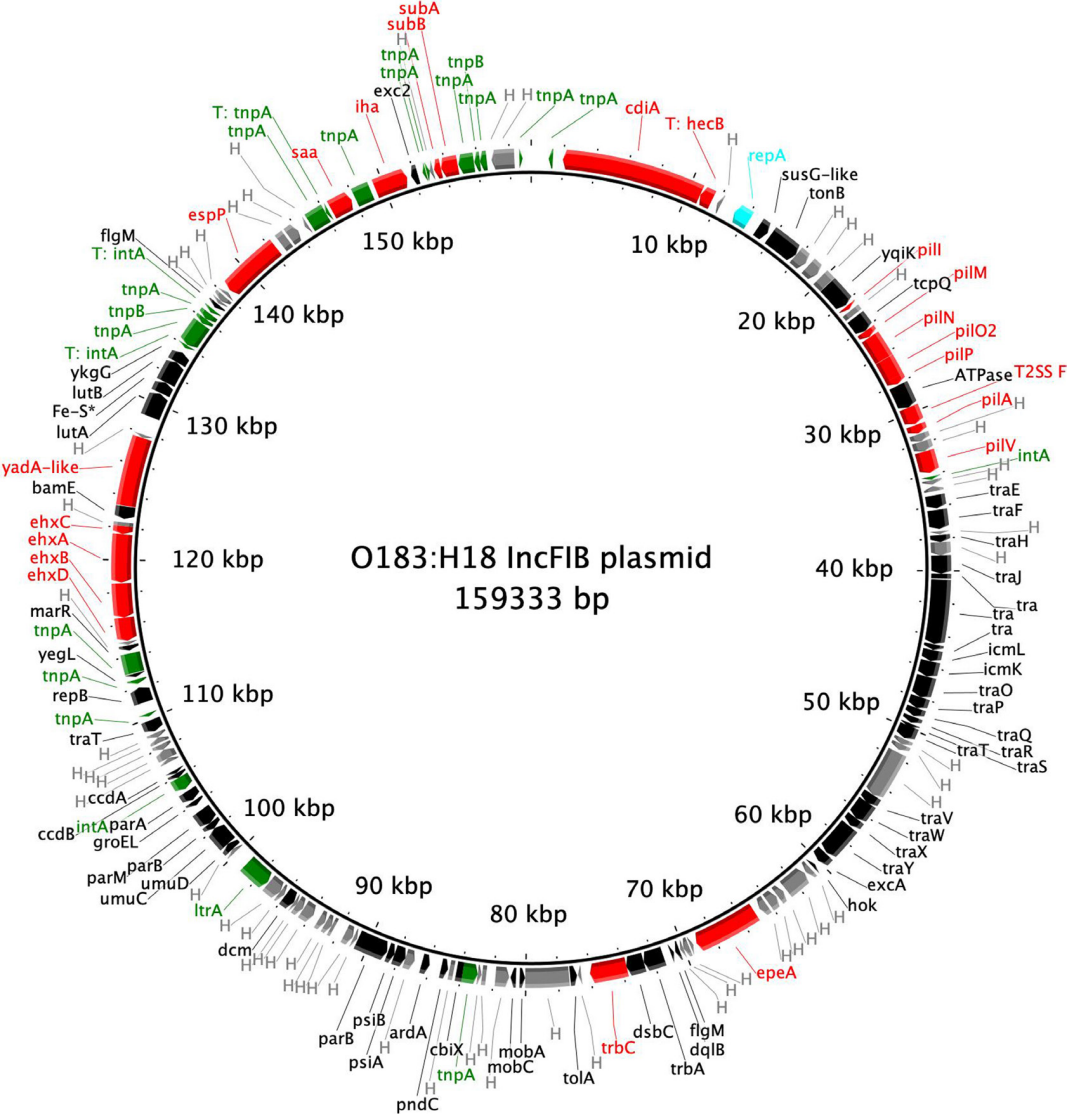
BRIG plot showing the gene content of the 159kbp IncFIB plasmids detected in outbreak strains in this study. Virulence genes are coloured in red, genes related to mobilisation are in green and *repA* labelled in blue.

To contextualise the UKHSA sequencing data, 53 isolates belonging to ST657 from global sources were added to phylogeny. The majority of the human isolates from the UKHSA archive were located on the same branch that split into two clades (Fig. S1). The remaining sporadic UK isolates were found interspersed with isolates from across the globe and from a variety of animal and environmental sources (Fig. S1). The putative virulence genes *espP*, *ehxA, saa, ihA and subA* were plasmid encoded (Fig. 5). The plasmid was conserved across all the isolates linked to the outbreak. For the most part, the acquisition of *stx* across the global phylogeny appears to correspond with the acquisition of the plasmid-encoded putative virulence genes *espP*, *ehxA, saa, ihA* and *subA* (Fig. S1).

## Discussion

Here, we describe a foodborne outbreak caused by an atypical serotype of STEC, O183:H18. There are few reports of STEC O183:H18 in the literature. One exception is a report of an outbreak of human gastroenteritis in Japan caused by *Escherichia albertii* and STEC O183, where the authors concluded that *E. albertii* was the etiological agent [39]. In addition to belonging to a serotype rarely seen globally or in the UK, the outbreak strain was unusual as it did not have either of the established mechanisms that facilitate attachment of STEC to the human gut mucosa, evidenced by the absence of *eae* and *aggR*. To date, with the exception of six cases with epidemiological links to the outbreak of STEC O104:H4 in Germany in 2011 [40], all foodborne outbreaks of STEC in the UK have been caused by *eae*-positive strains (UKHSA in-house data). In addition to the presence of *stx2a*, known to be associated with progression to HUS [8], a review of the sequencing data also identified a plasmid carrying the putative virulence factors, *espP*, *ehxA, saa, ihA and subA*. Analysis of UKHSA surveillance data has highlighted that these putative virulence factors have been previously identified in STEC harbouring *stx* subtypes *stx1a, stx1c and stx2b*, and causing milder gastrointestinal symptoms than those patients linked to this outbreak [41]. However, the combined presence of *espP*, *ehxA, saa, ihA and subA* with *stx2a,* may have enhanced the pathogenic potential of this strain.

Previous studies have shown that analysis of the presence of *stx*-encoding bacteriophage across the population structure of *E. coli* O157:H7, reveals a dynamic and complex picture of acquisition and loss [17,42]. The emergence of each of the sub-lineages of STEC O157:H7 that have dominated the landscape at different times in England over the last 40 years (lineage I/II, and sub-lineages Ic, and IIb) was driven by the acquisition of a unique *stx2a*-encoding bacteriophage, suggesting independent acquisition from an external source, rather than intra-UK lineage to lineage phage transmission [17,43] . Analysis of the outbreak strain described here revealed that the *stx2a*-encoding prophage was unrelated to *stx*-encoding bacteriophage previously identified in the established UK STEC lineages. Instead, it was located on a sparsely populated branch of the UKHSA bacteriophage phylogeny comprising a diverse set of phages encoding a variety of *stx* subtypes. The *E. coli* hosting these phages themselves belonged to a variety of different clonal complexes and serotypes. More than half of the cases had travel related infections, although they reported travel to different countries. Currently, the meaningfulness of these observations is unclear, and we are exploring whether further analysis of the genetic structure of the *stx*-encoding bacteriophage could provide insight into the evolutionary history, origins and pathogenicity of emerging STEC serotypes [43,44]. STEC O183:H18 is a rare serotype in the UKHSA database, and together with our *stx*-encoding prophage analysis, we hypothesised that either this STEC serotype has been recently imported into the UK, or it is a domestic strain of *E. coli* that has recently acquired the *stx*-encoding phage from an external source.

The outbreak is likely to be more widespread than described here, as many local laboratories in England do not perform PCR and would therefore miss the diagnosis. In 2023, approximately one third of local and regional hospital laboratories in England have implemented PCR, although none of these laboratories have the capability to culture all STEC serotypes (UKHSA in-house data). Comprehensive culture of non-O157 STEC requires referral to the Gastrointestinal Bacteria Reference Unit at UKHSA [45]. The STEC Operational Guidance recommends referral of faecal specimens to GBRU when HUS is suspected and for cases reporting severe gastrointestinal symptoms (Public health operational guidance for Shiga-toxin producing *Escherichia coli* [STEC] including STEC O157 and non-O157 infections [publishing.service.gov.uk]). However, compliance with the guidelines is inconsistent and we predict that case ascertainment during this outbreak was likely to have been poor.

Although the epidemiological analysis implicated meat products made from beef mince as a potential vehicle, the complexity of the beef supply lines confounded the food chain investigations (Case Study: The Beef Supply Chain [futurelearn.com]). Beef can be farmed from beef or dairy bred cattle, and the animals may move through growing and finishing stages on multiple farms prior to slaughter. Cattle are sold to processors either directly, via auction or to third party agents. Animals may be processed into a variety of products at a variety of different facilities which are then sold to multiple retailers, wholesalers and food service markets. Once the outbreak was over, it was challenging for partner agencies to justify continuation of resource intense food chain investigations, and so on this occasion full trace-back was not possible.

*E. coli* are ubiquitous in the environment and animal reservoir and have a dynamic genome capable of acquiring and maintaining mobile genetic elements, such as bacteriophage [1]. Bacteriophage encoding the *stx* genes are abundant in the environment and there is evidence of continued acquisition, loss and reacquisition of *stx*-encoding bacteriophage across the *E. coli* population structure [17,42, 43], . If conditions are right, the *stx* phage is incorporated into the genome, and in certain clones becomes stable and the newly emerged STEC clone persists becoming established as a cause of human clinical disease [1]. Other STEC clones may emerge and cause an outbreak following a combination of events creating a transient perfect storm, but then do not become endemic in the environment or animal reservoir, and persistence is not maintained by person-to-person transmission. The acquisition of *stx* appeared to correspond with the presence of the plasmid encoded virulence factors *subA*, *ehxA*, *saa,* and *lpfA*. As previously described, we considered the possibility that the *stx*-encoding phage may have been acquired on the plasmid and then subsequently incorporated onto the chromosome [46].

At UKHSA we have been using short read WGS for outbreak detection and investigation for over a decade, and more recently we have been exploring the utility of long read sequencing for public health surveillance. During this outbreak, we used long read sequencing to characterise the plasmid and prophage content to look for evidence to explain the emergence of the novel, atypical strain of STEC. Although we were unable to determine source and transmission route of the outbreak strain, the genomic analysis revealed potential clues as to how novel strains for STEC evolve. Whether or not STEC O183:H18 will emerge as an on-going threat to public health remains to be seen. However, with the implementation of PCR capable of detecting all STEC, and genome sequencing for typing and virulence profiling, we have the tools to enable us to monitor the changing landscape of STEC. Improvements in the standardised collection of epidemiological data and trace-back strategies within the food industry, will ensure we have a surveillance system capable of alerting us to emerging threats to public health.

## supplementary material

10.1099/mgen.0.001243 Uncited Fig. S1.

10.1099/mgen.0.001243 Uncited Table S1.

## Abbreviations

BWA: burrows-wheeler alignment
CTSMAC: cefixime tellurite sorbitol MacConkey
EAEC: enteroaggregative *Escherichia coli*
EIEC: enteroinvasive *Escherichia coli*
EPEC: enteropathogenic *Escherichia coli*
ESQ: enhanced surveillance questionnaire
ETEC: enterotoxigenic *Eschichia coli*
GBRU: Gastrointestinal Bacteria Reference Unit
HUS: haemolytic uraemic syndrome
IMT: incident management team
LEE: locus enterocyte effacement
MLST: multilocus sequence type
MOST: metric orientated sequence typer
NCBI: National Center for Biotechnology Information
NESSS: National Enhanced STEC Surveillance System
SERL: Scottish E. coli Reference Laboratory
SNP: single nucleotide polymorphism
SRA: sequence read archive
STEC: Shiga toxin-producing *Escherichia coli*
stx: shiga toxin
UKHSA: UK Health Security Agency
WGS: whole genome sequencing
