# Supplementary figures and images for: Genomic analysis of an outbreak of Shiga toxin-producing Escherichia coli O183:H18 in the United Kingdom, 2023
Source: Microb Genom. 2024 May 21;10(5):001243. doi: 10.1099/mgen.0.001243 (PMC11165631; doi:10.1099/mgen.0.001243)

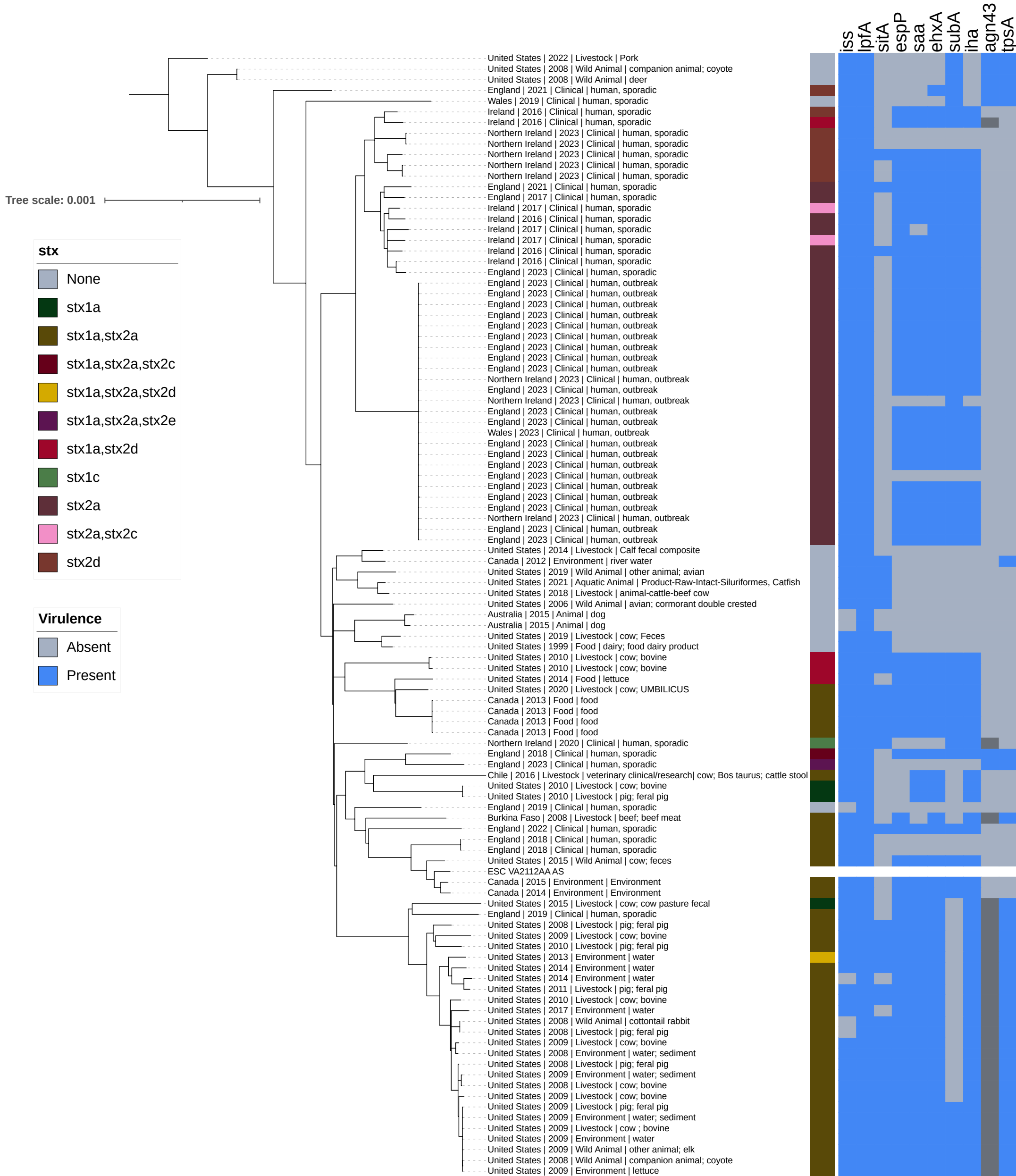

Supplement: Uncited Fig. S1. [file mgen-10-01243-s001.pdf]
